# Dietary plant extracts modulate gene expression profiles in alveolar macrophages of pigs experimentally infected with porcine reproductive and respiratory syndrome virus

**DOI:** 10.1186/s40104-020-00475-w

**Published:** 2020-07-14

**Authors:** Kwangwook Kim, Peng Ji, Minho Song, Tung M. Che, David Bravo, James E. Pettigrew, Yanhong Liu

**Affiliations:** 1 Department of Animal Science, University of California, Davis, CA USA; 2 Department of Nutrition, University of California, Davis, CA USA; 3 Department of Animal Science and Biotechnology, Chungnam National University, Daejeon, South Korea; 4 Department of Animal Production, Nong Lam University, Ho Chi Minh City, Vietnam; 5 Pancosma SA, Geneva, Switzerland; 6 Current address: Land O’Lakes Inc., Arden Hills, MN USA; 7 Department of Animal Science, University of Illinois, Urbana, IL USA

**Keywords:** Alveolar macrophages, Gene expression, Plant extracts, PRRSV, Weaned pigs

## Abstract

**Background:**

Our previous study showed that 3 plant extracts enhanced the immune responses and growth efficiency of weaned pigs infected with porcine reproductive and respiratory syndrome virus (PRRSV), which is one of the most economically important disease in swine industry. However, each plant extract differently effected on growth efficiency and immune responses. Therefore, the objective of this study was conducted to characterize the effects and investigate the potential underlying mechanisms of 3 plant extracts on gene expression of alveolar macrophages in weaned pigs experimentally infected with PRRSV.

**Results:**

PRRSV infection altered (*P* < 0.05) the expression of 1,352 genes in pigs fed the control (CON; 755 up, 597 down). Compared with the infected CON, feeding capsicum (CAP), garlic botanical (GAR), or turmeric oleoresin (TUR) altered the expression of 46 genes (24 up, 22 down), 134 genes (59 up, 75 down), or 98 genes (55 up, 43 down) in alveolar macrophages of PRRSV-infected pigs, respectively. PRRSV infection up-regulated (*P* < 0.05) the expression of genes related to cell apoptosis, immune system process, and response to stimulus, but down-regulated (*P* < 0.05) the expression of genes involved in signaling transduction and innate immune response. Compared with the infected CON, feeding TUR or GAR reduced (*P* < 0.05) the expression of genes associated with antigen processing and presentation, feeding CAP up-regulated (*P* < 0.05) the expression of genes involved in antigen processing and presentation. Supplementation of CAP, GAR, or TUR also enhanced (*P* < 0.05) the expression of several genes related to amino acid metabolism, steroid hormone synthesis, or RNA degradation, respectively.

**Conclusions:**

The results suggest that 3 plant extracts differently regulated the expression of genes in alveolar macrophages of PRRSV-infected pigs, especially altering genes involved in immunity.

## Background

The porcine reproductive and respiratory syndrome virus (PRRSV) is a highly prevalent disease in swine herds. The characteristics of this syndrome are reproductive failure in late-term gestation in sows, respiratory disease in pigs of all ages, decreased performance, and increased mortality [1–3]. The virus for this syndrome, PRRSV, has a complex interaction with the immune system because the primary targets of PRRSV are mononuclear phagocytic cells, i.e., macrophages, monocytes, and dendritic cells located in the mucosal surface of the respiratory tract [4]. The PRRSV infection can induce the release of copious amounts of inflammatory cytokines which are known to cause tissue damage and systemic illness, including fever and lethargy [5–7].

Our previously published study reported that feeding 10 mg/kg of capsicum oleoresin, garlic botanical, or turmeric oleoresin reduced viral load in pigs infected with PRRSV on d 7 and 14 post-inoculation [7]. Feeding these plant extracts also inhibited the increase in serum pro-inflammatory cytokines (TNF-α or IL-1β) and an acute phase protein (C-reactive protein), but enhanced a serum anti-inflammatory cytokine and the population of B lymphocytes and cytotoxic T cells in the blood of PRRSV-infected pigs [7]. These results indicate supplementation of plant extracts strengthened immune responses of pigs and suppresses ongoing inflammation, which may prevent secondary infections of pigs. Consequently, lower rectal remperature and better feed efficiency was also observed in PRRSV-infected pigs when they were supplemented with plant extracts [7]. In this study, capsicum and turmeric are extracted oleoresins, which were standardized to 6% capsaicin and dihydrocapsaicin, and 98% curcuminoides, respectively. Garlic botanical is extracted from garlic, standardized to 40% propyl thiosulfonates. Low dose (10 mg/kg) of plant extracts was used to test dietary supplememtation of plant extracts on immune responses of pigs under disease challenge conditions, rather than their antimicrobial effects. The 3 plant extracts tested showed different effects in pig immunity, suggesting they may work through different mechanisms. Alveolar macrophages play critical roles in the lung immunity, and are the cells preferentially infected by PRRSV. Therefore, the objective of this study was to characterize gene expression in alveolar macrophages of pigs as affected by PRRSV infection and by plant extracts, using the Affymetrix Genechip Porcine Genome Array followed by real-time PCR (qRT-PCR) validation.

## Materials and methods

### Animals, housing, experimental design, and diet

A total of 64 weaned piglets (G performer × Fertilis 25; Genetiporc Inc., Alexandria, MN) with the same number of gilts and barrows and 7.8 ± 0.3 kg of initial BW were selected from the Swine Research Center of the University of Illinois at Urbana-Champaign. The piglets used in this experiment were verified PRRSV-free. After weaning, all pigs were transferred to the disease containment chambers and randomly assigned to treatment in a randomized complete block design with weight within sex as the blocks and pig as the experimental unit. Pigs were housed in individual pens for 28 d (14 d before and 14 d after the PRRSV challenge). There were 2 suites of 8 chambers, and each suite was used for either PRRSV-challenged or unchallenged pigs. There was a total of 64 individual pens with 4 pens in each of 16 chambers. The piglets had ad libitum access to feed and water.

The treatments were in a 2 × 4 factorial arrangement (with or without PRRSV challenge; 4 different dietary treatments). There were 8 replicates per treatment. In the PRRSV challenge group, all pigs were intranasally inoculated with 2 mL of high-virulence strain of PRRSV (Purdue isolate P-129) containing 10^5^ of 50% tissue culture infective dose. In the unchallenged group, pigs were inoculated with 2 mL PBS as the sham control. The 4 dietary treatments were the complex nursery basal diet (CON) and the addition of 10 mg/kg capsicum oleoresin (CAP), 10 mg/kg garlic botanical (GAR), or 10 mg/kg turmeric oleoresin (TUR) to the CON respectively and fed throughout the experiment (28 d). All 3 plant extracts were obtained from Pancosma S. A. (Geneva, Switzerland). Capsicum and turmeric are extracted oleoresins, which were standardized to 6% capsaicin and dihydrocapsaicin, and 98% curcuminoides, respectively. Garlic botanical is extract from garlic, standardized to 40% propyl thiosulfonates. The CON diet was formulated to meet or exceed the NRC [8] estimates of nutrient requirements of weaned pigs (Table 1). Spray-dried plasma, antibiotics, or zinc oxide were not included in the CON diet. The same experimental diets were fed throughout the experiment.

**Table 1:** Ingredient composition of basal diet (as-fed basis)

Ingredient	Amount, %
Corn, ground	41.54
Whey, dried	15.00
Soybean meal, dehulled	10.82
Fishmeal, Menhaden	10.00
Lactose	10.00
Soy protein concentrate	5.00
Poultry byproduct meal	4.27
Soybean oil	2.67
Mineral premix^a^	0.35
Vitamin premix^b^	0.20
*L*-Lysine∙HCl	0.05
*DL*-Met	0.05
*L*-Thr	0.03
*L*-Trp	0.02
Total	100.00
Calculated energy and nutrients	
ME, kcal/kg	3,480
CP, %	22.67
Fat, %	6.34
Ca, %	0.80
P, %	0.72
Available P, %	0.49
Lys, %	1.50
Lactose, %	21.00

^a^Provided per kg of diet: 3,000 mg of NaCl; 100 mg of Zn from zinc oxide; 90 mg of Fe from ferrous sulfate; 20 mg of Mn from manganese oxide; 8 mg of Cu from copper sulfate; 0.35 mg of I from calcium iodide; 0.30 mg of Se from sodium selenite

^b^Provided per kg of diet: 2,273 μg of retinyl acetate; 17 μg of cholecalciferol; 88 mg of *DL*-α-tocopheryl acetate; 4 mg of menadione from menadione sodium bisulfite complex; 33 mg of niacin; 24 mg of *D*-Ca-pantothenate; 9 mg of riboflavin; 35 μg of vitamin B_12_; 324 mg of choline chloride

### Sample collection

A total of 32 pigs (4 pigs from each treatment, 2 males and 2 females) were euthanized at d 14 post-inoculation (PI). Before being euthanized, pigs were anesthetized by intramuscular injection of a 1-mL combination of telazol, ketamine, and xylazine (2:1:1) per 23 kg BW. The final mixture contained 100 mg telazol, 50 mg ketamine, and 50 mg xylazine in 1 mL (Fort Dodge Animal Health, For Dodge, IA, USA). After anesthesia, pigs were euthanized by intracardiac injection with 78 mg sodium pentobarbital (Sleepaway) per 1 kg of BW (Henry Schein, Inc., Indianapolis, IN, USA). Porcine alveolar macrophages were collected from bronchoalveolar lavage based on the procedures in Liu et al. [9]. Briefly, lungs with intact trachea were removed immediately after euthanizing pigs and 100 mL PBS was poured into them through the trachea. After massaging the lungs about 60 s, the lavage fluid was filtered through a double layer of sterile gauze into 50 mL conical centrifuge tubes and then pelleted by centrifuging at 400×g for 15 min at room temperature. The pelleted cells were washed twice with PBS and immediately stored in liquid nitrogen for further analysis.

### Total RNA extraction and gene expression by microarrays

Total RNA (4 pigs/treatment) from alveolar macrophages isolated at d 14 PI was extracted using PureLink RNA Micro Kit according to the manufacturer’s instructions (Invitrogen, Carlsbad, CA, USA). The RNA quality and quantity were assessed using the Agilent 2100 Bioanalyzer (Agilent Technologies, Santa Clara, CA, USA) and the ND-1000 Nanodrop spectrophotometer (Thermo Scientific, Wilmington, DE), respectively. All samples used for further analysis had an OD_260_/OD_280_ ratio of 1.9 to 2.1, an OD_260_/OD_230_ ratio of > 1.9, and an RNA integrity number of ≥9.0. Double-stranded cDNA was first synthesized and employed as a template for *in vitro* amplification and labeling by using GeneChip Expression 3′-Amplification IVT Labelling Kit (Affymetrix Inc., Santa Clara, CA, USA). Then, cDNA was used to synthesize cRNA which was hydrolyzed to produce fragmented cRNA in the 35–200 nucleotide size range for proper hybridization. The fragmented cRNA was labeled and further hybridized to the Affymetrix GeneChip Porcine Genome Array (Affymetrix Inc., Santa Clara, CA, USA). Each array consists of 23,937 probe sets to interrogate 23,256 transcripts in the pig genome, which represents 20,201 genes. Thirty-two chips in total were used in this experiment.

### Analysis of microarray data

All quality control assessments, data processing, and statistical analysis were done in R (R Development Core Team, 2008) using packages from the Bioconductor project [10] as indicated below. Quality control assessment [11] showed that all arrays were of acceptable quality. The arrays were processed with the guanine cytosine robust multi-array analysis algorithm, which performs a guanine-cytosine-based background-correction, does a quantile normalization between arrays, and summarizes the multiple probes into a single probe set value using a median polish algorithm [12].

Testing for differential gene expression was done by fitting a mixed linear model equivalent to a 2 × 4 factorial ANOVA using the limma package [6], which uses an empirical Bayes correction that helps to improve power by borrowing information across genes [13]. The statistical model included effects of PRRSV challenge, diet, and their interaction as fixed effects and block as random effect. The appropriate pairwise comparisons were fit as contrasts from the model. The following 4 comparisons were of interest and presented in the current manuscript: Infected control (ICON) vs. CON, infected capsicum oleoresin (ICAP) vs. ICON, infected garlic botanical (IGAR) vs. ICON, and infected turmeric oleoresin (ITUR) vs. ICON. A total of 23,937 gene probe sets were included in the porcine array, but only 15,036 probe sets were detected in the alveolar macrophage. The limma model was fit and *P*-values were calculated using all 15,036 probe sets on the array. The modulated genes were defined by 1.5-fold difference and a cut-off of *P* < 0.05 by parameter tests.

### Bioinformatics analysis

A bioinformatics resources (DAVID Bioinformatics Resources 6.7; National Institute of Allergy and Infectious Diseases, NIH, Bethesda, MD; http://david.abcc.ncifcrf.gov) consists of an integrated biological knowledge base and analytic tools used to systematically extract biological meaning from large gene lists [14–16]. In brief, the analysis of selected genes in DAVID was as follows. First, all 15,036 probe sets in the porcine genome array (Affymetrix GeneChip Porcine Genome Array, Affymetrix Inc.) were submitted to DAVID and 4,073 of them were mapped with identified gene functions. These 4073 genes’ Entrez [National Center for Biotechnology Information (NCBI), USHHS, Washington, DC] identifications were uploaded as background for the analysis. Second, the modulated genes with 1.5-fold difference and a cut-off of *P* < 0.05 in each comparison were uploaded as the tested gene list. Third, the parameters and sub-parameters of interest in this experiment were established. In the present analysis, the main parameters in DAVID included gene ontology and pathways. The sub-parameter under gene ontology was gene ontology for biological process, cellular component, and molecular function, while the sub-parameter under pathways was Kyoto Encyclopedia of Genes and Genomes (KEGG) pathways. Finally, the functional annotation chart, which provided typical gene-term enrichment analysis, was run. The expression analysis systemic explorer (EASE) score, a modified Fisher Extract *P*-value, was used to examine the significance of gene-term enrichment. The EASE score < 0.05 was considered as significantly affected.

### Quantitative real-time PCR

The same total RNA (4 pigs/treatment) from alveolar macrophages used to run the microarray was also employed for qRT-PCR. First-Strand cDNA was produced from 1 μg of total RNA per sample (SuperScript III First-Strand Synthesis SuperMix for qRT-PCR; Invitrogen) in a total volume of 20 μL. Total RNA was denatured at 65 °C for 5 min and immediately annealed on ice for at least 1 min. Then, the reverse transcription reaction was performed at 50 °C for 50 min, followed by heat inactivation at 85 °C for 5 min.

To verify the results from the microarray, quantitative analysis of caspase 3 (*CASP3*)*,* chemokine ligand 5 (*CCL5***)***,* interferon gamma (*IFNG*), IL-1α (*IL1A*)*,* and IL-7 (*IL7*) in alveolar macrophages were assayed by qRT-PCR. Data normalization was accomplished using β-actin (*ACTB*) and glyceraldehyde 3-phosphate dehydrogenase (*GAPDH*) as housekeeping genes. Primers (Additional file 1: Supplementary Table 1) were designed based on published sequences in pigs using the NCBI (USHHS) online primer design tool and published literature, and synthesized commercially (Applied Biosystems, Foster, CA). One hundred nanograms of total RNA were assayed for each sample in triplicate. Each PCR reaction consisted of 5 μL of mixture (SYBR Green PCR Master Mix; Applied Biosystems), 0.4 μL of 10 μmol/L forward primer, 0.4 μL of 10 μmol/L reverse primer, 0.2 μL of DNase/RNase free water, and 4 μL of diluted cDNA. The qRT-PCR analysis was done using ABI PRISM 7900 Sequence Detection System (Applied Biosystems). Thermal cycling conditions were 50 °C for 2 min and 95 °C for 10 min, followed by 40 cycles with 15 s at 95 °C and 1 min at 60 °C. The dissociation cycle was 95 °C for 15 s plus 65 °C for 15 s. Standard curves were generated using serial dilutions of pooled cDNA from all samples. The arbitrary values were calculated based on the standard curve and normalized using the housekeeping genes.

## Results

### Gene expression profiles induced by PRRSV and plant extracts

PRRSV infection altered (*P* < 0.05) the expression of 1,352 (755 up-regulated, 597 down-regulated) genes in alveolar macrophages when ICON was compared with the uninfected CON (Table 2). Supplementation of plant extracts displayed different effects on the gene expression in alveolar macrophages of PRRSV-infected pigs. Compared with the ICON, CAP-fed pigs had altered (*P* < 0.05) expression of 46 genes with 24 up-regulated and 22 down-regulated genes, feeding GAR altered (*P* < 0.05) expression of 134 genes (59 up-regulated and 75 down-regulated), and TUR-fed pigs exhibited (*P* < 0.05) 98 changed genes (55 up-regulated and 43 down-regulated). All these altered genes were mapped in DAVID Bioinformatics Resources.

**Table 2:** Gene expression profiles changed by porcine reproductive and respiratory syndrome virus (PRRSV) infection and by dietary supplementation of plant extracts to PRRSV-infected pigs^a^

Dietary supplement	Comparison^b^	Up-regulated	Down-regulated	Total
PRRSV	ICON vs. CON	755	597	1,352
Capsicum oleoresin, 10 mg/kg	ICAP vs. ICON	24	22	46
Garlic botanical, 10 mg/kg	IGAR vs. ICON	59	75	134
Turmeric oleoresin, 10 mg/kg	ITUR vs. ICON	55	43	98

^a^The gene expression changed by fold-change cutoff of 1.5 and a *P*-value cutoff of 0.05. All data were analyzed by DAVID Bioinformatics Resources 6.7 (National Institute of Allergy and Infectious Diseases, NIH, Bethasda, MD, USA)

^b^CON = uninfected control diet-fed pigs; ICAP = infected capsicum oleoresin-fed pigs; ICON = infected control diet-fed pigs; IGAR = infected garlic botanical-fed pigs; ITUR = infected turmeric oleoresin-fed pigs

### Differential immune gene expression in alveolar macrophage of pigs

**Fig. 1:**
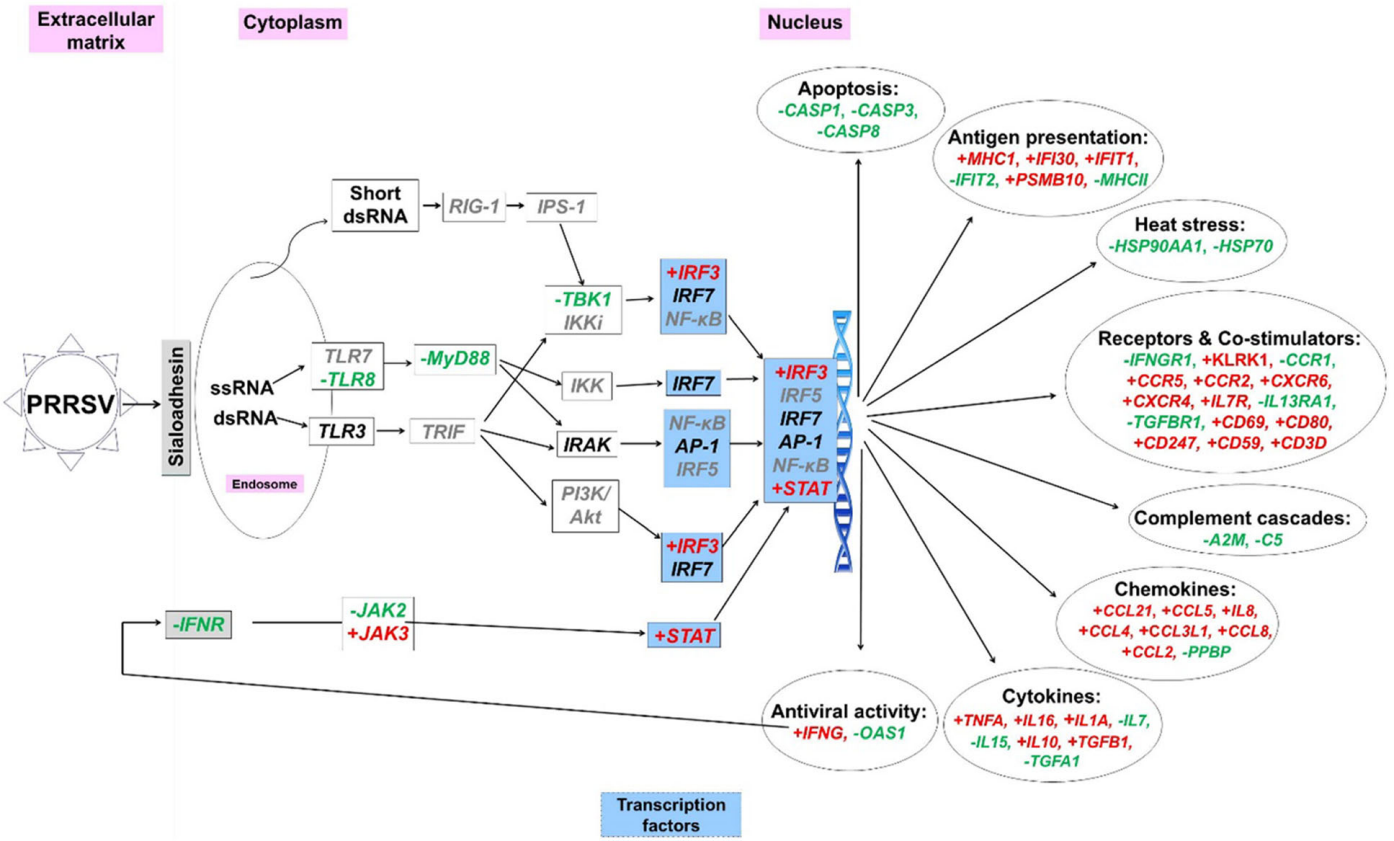
Porcine reproductive and respiratory syndrome virus (PRRSV) infection and the host’s immune responses. The bolded (+) signs and bolded (−) signs represent genes upregulated and downregulated, respectively, when pigs fed with control diet. Genes with gray color did not have information from the gene chip (Affymetrix Inc., Santa Clara, CA, USA), and genes with black color were not affected by PRRSV at d 14 post inoculation. A2M: alpha-2-macroglobulin; Akt: protein kinase B; AP-1: activator protein 1; C5: complement component 5; CASP: caspase, apoptosis-related cysteine peptidase; CCL: C-C motif ligand; CCR: chemokine (C-C motif) receptor; CD: cluster of differentiation; CXCR: chemokine (C-X-C motif) receptor; HSP: heat shock protein; IFI30: interferon, gamma-inducible protein 30; IFIT: interferon-induced protein with tetratricopeptide; IFN: interferon; IFNGR: interferon gamma receptor 1; IKK: IkB kinase; IKKi: inducible IkB kinase; IL7R: interleukin 7 receptor; IL13RA1: interleukin 13 receptor, alpha 1; IPS-1: an adaptor triggering RIG-1; IRAK: interleukin-1 receptor-associated kinase; IRF: interferon regulatory factor; JAK: Janus kinase; KLRK: killer cell lectin-like receptor subfamily K; MHC: major histocompatibility complex; MyD88: myeloid differentiation factor 88; NF-κB: nuclear factor-κB; OAS1: 2′-5′-oligoadenylate synthetase 1; PI3K: phosphoinositide-3-kinase; PPBP: pro-platelet basic protein; PSMB10: proteasome subunit beta 10; RIG: retinoic acid-inducible gene; STAT: signal transducers and activators of transcription; TBK1: TANK-binding kinase; TGF: transforming growth factor; TLR: toll like receptor; TNFA: tumor necrosis factor-α. Feeding plant extracts had several effects counter (*P* < 0.05) to those of PRRSV. Capsicum oleoresin: *A2M, CCL21, CCR2, CD3D, HSP70, HSP90AA1*, and *IL1A*; Garlic botanical: *C5, CCL21, HSP70, HSP90AA1, IFIT1, IFIT2, IL1A, MHC1*, and *PPBP*; Turmeric oleoresin: *C5, CASP3, CASP8, CCL21, IFI30, IFIT2, IL1A, JAK2, PSMB10*, and *TLR8*. See online version for figure in color

PRRSV infection modulated (*P* < 0.05) the mRNA expression of genes related to innate immune responses in alveolar macrophages of weaned pigs (Fig. 1). PRRSV infection altered (*P* < 0.05) the expression level of genes related to cell apoptosis (decrease in *CASP1*, *CASP3*, and *CASP8*), antigen presentation (increase in *MHC1*, *IFI30*, *IFIT1* and *PSMB10*, and decrease in *IFIT2* and *MHCII*), heat stress (decrease in *HSP90AA1* and *HSP70*), receptors and co-stimulators (increase in *KLRK1, CCR5, CCR2, CXCR6, CXCR4, IL7R, CD69, CD80, CD247, CD59*, and *CD3D*, and decrease in *IFNGR1, CCR1, IL13RA1*, and *TGFBR1*), complement cascades (decrease in *A2M* and *C5*), chemokines (increase in *CCL2, CCL3L1, CCL4, CCL5, CCL21*, and *IL8*, and decrease in *PPBP*), cytokines (increase in *IL1A, IL10, IL16, TGFB1*, and *TNFA*, and decrease in *IL7, IL15*, and *TGFA1*), and antiviral activity (increase in *IFNG* and decrease in *OAS1*). PRRSV infection also up-regulated (*P* < 0.05) the expression of several genes associated with transcriptional factors (*IRF3* and *STAT*).

The 3 plant extracts had different impacts on expression of genes that were counter to the effects of PRRSV. Feeding CAP counteracted (*P* < 0.05) effects of PRRSV on *A2M, CCL21, CCR2, CD3D, HSP70, HSP90AA1*, and *IL1A*. Feeding GAR counteracted (*P* < 0.05) the disease effects on *C5, CCL21, HSP70, HSP90AA1, IFIT1, IFIT2, IL1A, MHC1*, and *PPBP*. Feeding TUR reversed (*P* < 0.05) the effects of PRRSV on the expression of *C5, CASP3, CASP8, CCL21, IFI30, IFIT2, IL1A, JAK2, PSMB10*, and *TLR8*.

### Biological process analysis

**Fig. 2:**
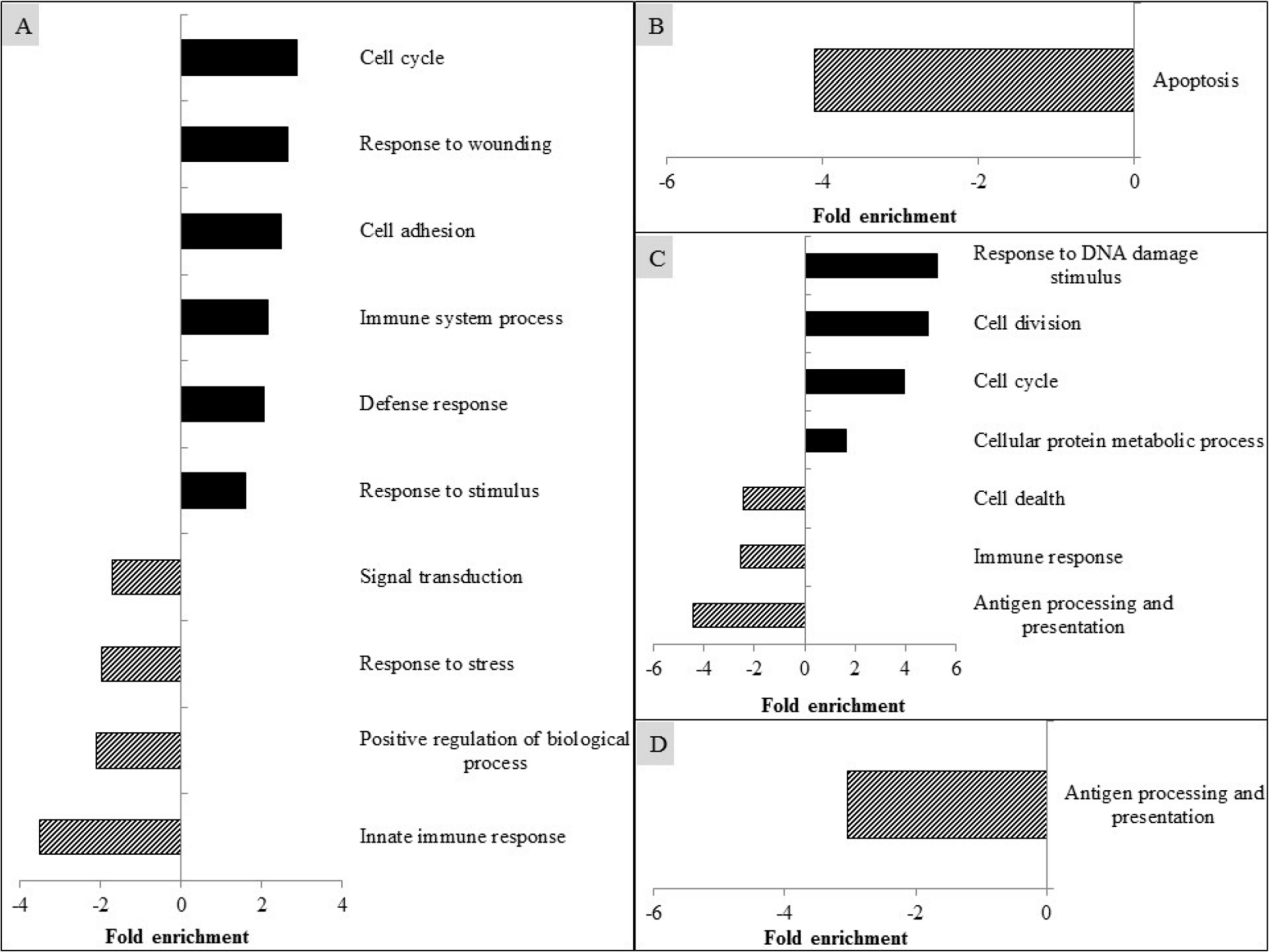
Modulation of biological process in alveolar macrophages of pigs by PRRSV infection (**a**), and supplementation of capsicum oleoresin (CAP; **b**), garlic botanical (GAR; **c**), or turmeric oleoresin (TUR; **d**)

The altered genes as analyzed by the gene ontology biological process using DAVID are presented in Fig. 2 and Additional file 1: Supplementary Tables 2 to 5. PRRSV infection up-regulated (EASE score < 0.05) the expression of genes related to cell cycle, response to wounding, cell adhesion, immune system process, defense response, and response to stimulus, but down-regulated (EASE score < 0.05) the expression of genes in the biological processes of signaling transduction, response to stress, positive regulation of biological process, and innate immune response (Fig. 2a). Compared with the ICON, supplementation of CAP down-regulated (EASE score < 0.05) gene expression of apoptosis (Fig. 2b) and inclusion of TUR reduced (EASE score < 0.05) the expression of genes related to antigen processing and presentation (Fig. 2d). Addition of GAR increased (EASE score < 0.05) the biological processes related to response to DNA damage stimulus, cell division, cell cycle, and cellular protein metabolic process, but decreased (EASE score < 0.05) the expression of genes associated with cell death, immune response, and antigen processing and presentation (Fig. 2c).

### Cellular component analysis

**Fig. 3:**
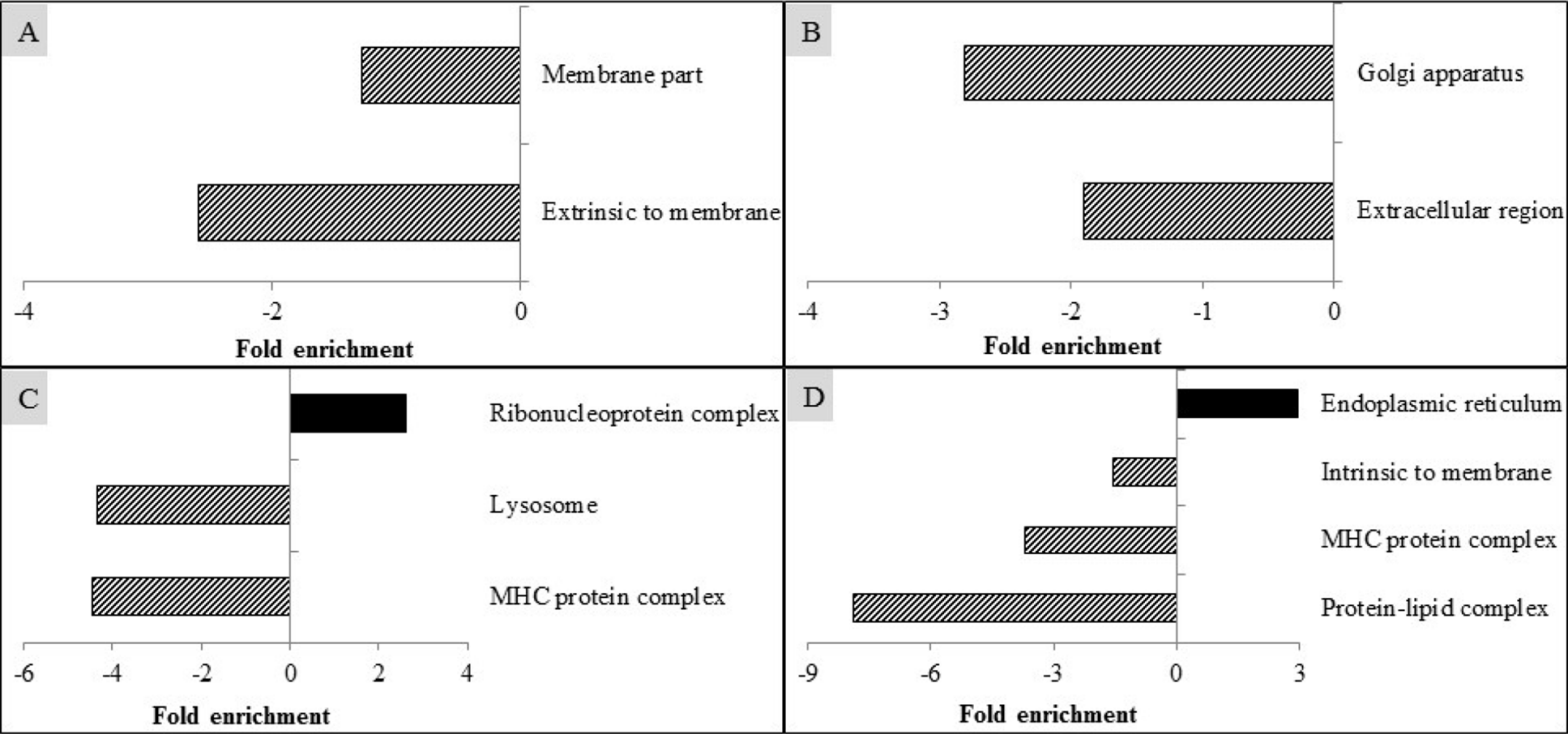
Modulation of cellular component in alveolar macrophages of pigs by PRRSV infection (**a**), and by supplementation of capsicum oleoresin (CAP; **b**), garlic botanical (GAR; **c**), or turmeric oleoresin (TUR; **d**)

The altered genes as analyzed by the gene ontology cellular component using DAVID are presented in Fig. 3. Compared with the CON, PRRSV infection down-regulated (EASE score < 0.05) the expression of genes related to cellular component in membrane part and extrinsic to membrane (Fig. 3a). Compared with the ICON, supplementation of CAP decreased (EASE score < 0.05) cellular component in Golgi apparatus and extracellular region (Fig. 3b), supplementation of GAR increased (EASE score < 0.05) ribonucleoprotein complex but decreased (EASE score < 0.05) cellular components associated with lysosome and MHC protein complex (Fig. 3c). The inclusion of TUR up-regulated (EASE score < 0.05) the expression of genes related to cellular component of endoplasmic reticulum, but down-regulated (EASE score < 0.05) the expression of genes associated with intrinsic to membrane, MHC protein complex, and protein-lipid complex (Fig. 3d).

### Molecular function analysis

**Fig. 4:**
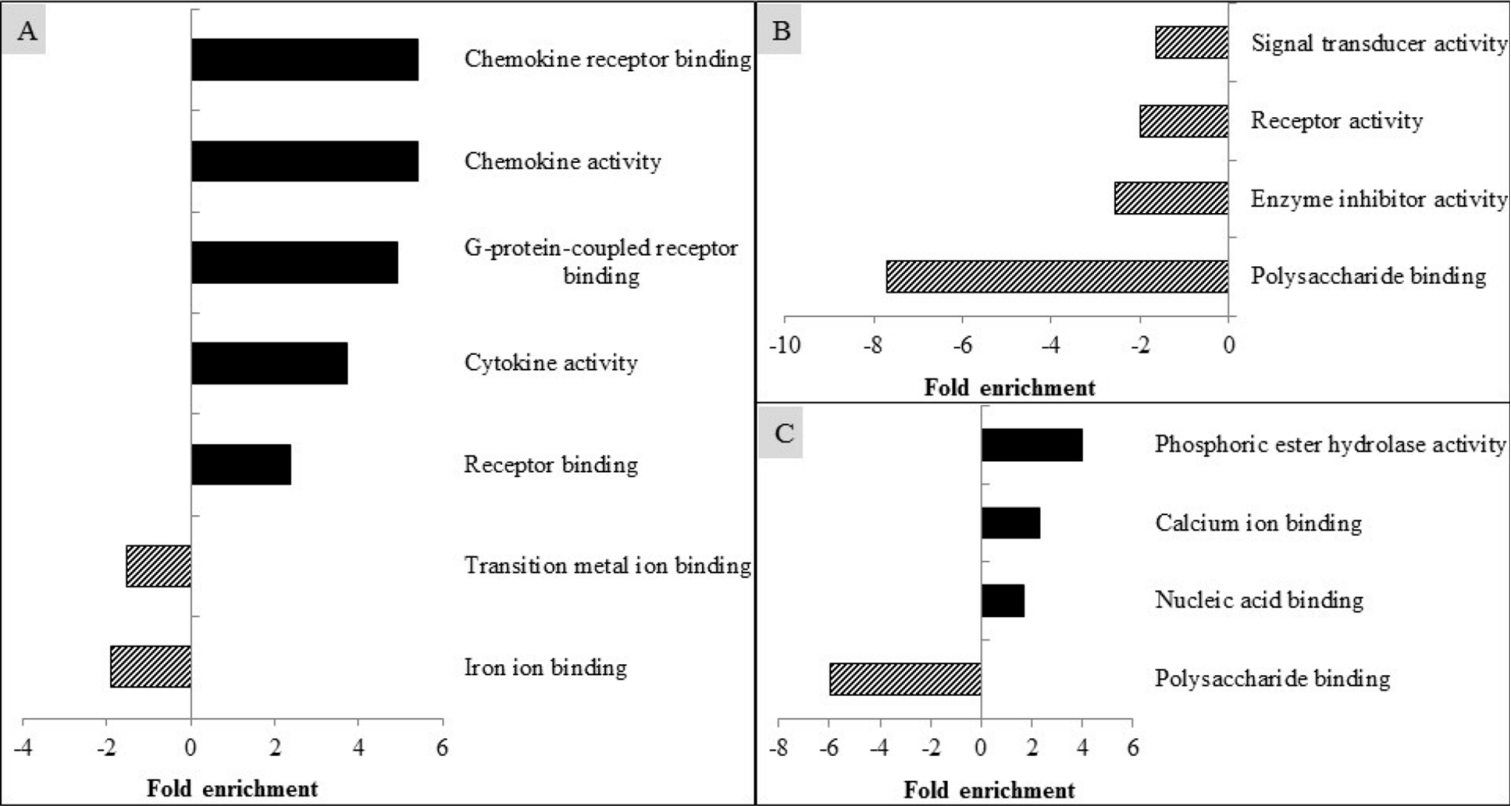
Modulation of molecular function in alveolar macrophages of pigs by PRRSV infection (**a**), and by supplementation of garlic botanical (GAR; **b**), or turmeric oleoresin (TUR; **c**)

The altered genes were also analyzed by the gene ontology molecular function using DAVID and presented in Fig. 4. PRRSV infection up-regulated (EASE score < 0.05) the expression of genes associated with the cellular function of chemokine receptor binding, chemokine activity, G-protein-coupled receptor binding, cytokine activity, and receptor binding, but down-regulated (EASE score < 0.05) transition metal ion binding and iron ion binding (Fig. 4a). No differences were observed in supplementation of CAP on gene expression related to molecular function compared with ICON. Inclusion of GAR down-regulated (EASE score < 0.05) the expression of genes associated with signal transducer activity, receptor activity, enzyme inhibitor activity, and polysaccharide binding, compared with the ICON (Fig. 4b). Feeding TUR to PRRSV-infected pigs increased (EASE score < 0.05) gene expression in molecular functions of phosphoric ester hydrolase activity, calcium ion binding, and nucleic acid binding, but decreased (EASE score < 0.05) the expression of genes associated with polysaccharide binding (Fig. 4c).

### The KEGG pathway analysis

**Fig. 5:**
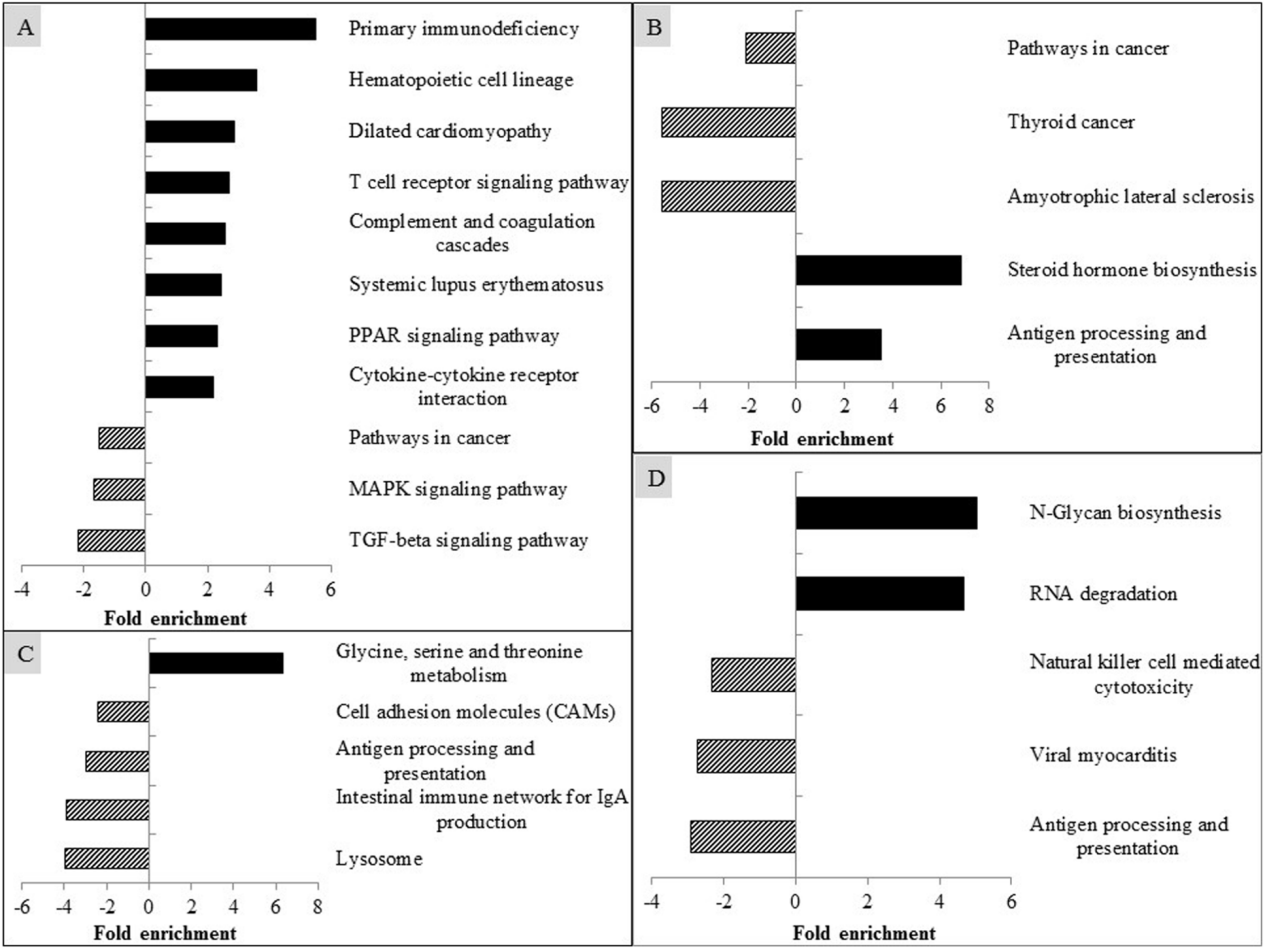
Modulation of Kyoto Encyclopedia of Genes and Genomes (KEGG) pathway in alveolar macrophages of pigs by PRRSV infection (**a**), and by supplementation of capsicum oleoresin (CAP; **b**), garlic botanical (GAR; **c**), or turmeric oleoresin (TUR; **d**)

Results of the KEGG pathway analysis are presented in Fig. 5 and Additional file 1: Supplementary Tables 2 to 5. Compared with the CON, PRRSV infection (EASE score < 0.05) up-regulated the expression of genes involved in the following pathways: primary immunodeficiency, hematopoietic cell lineage, dilated cardiomyopathy, T cell receptor signaling pathway, complement and coagulation cascades, systemic lupus erythematosus, PPAR signaling pathway, and cytokine-cytokine receptor interaction, but down-regulated (EASE score < 0.05) the expression of genes related to pathways in cancer, MAPK signaling pathway, and TGF-beta signaling pathway (Fig. 5a). Compared with the ICON, CAP increased (EASE score < 0.05) the gene expression related to steroid hormone biosynthesis and antigen processing and presentation, but decreased (EASE score < 0.05) the expression of genes involved in pathways in cancer, thyroid cancer, and amyotrophic lateral sclerosis (Fig. 5b). Supplementation of GAR up-regulated (EASE score < 0.05) glycine, serine, and threonine metabolism, but down-regulated (EASE score < 0.05) cell adhesion molecules, antigen processing and presentation, intestinal immune network for IgA production, and lysosome (Fig. 5c). Addition of TUR enhanced (EASE score < 0.05) the expression of genes related to N-glycan biosynthesis and RNA degradation, but reduced (EASE score < 0.05) gene expression in the pathways of natural killer cell mediated cytotoxicity, viral myocarditis, and antigen processing and presentation (Fig. 5d).

### Validation of gene expression by quantitative real-time PCR

**Fig. 6:**
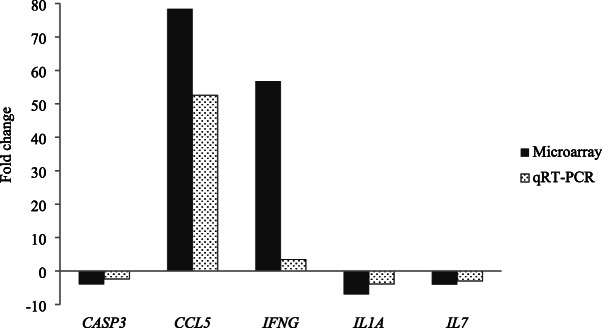
Verification of gene expression in alveolar macrophage by quantitative real-time PCR (qRT-PCR) by the fold change of porcine reproductive and respiratory syndrome virus infected control versus sham control. *CASP3* = caspase 3; *CCL5* = chemokine (C-C motif) ligand 5; *IFNG* = interferon-gamma, *IL1A* = IL-1α, *IL7* = IL-7

Five immune-related genes, *CASP3, CCL5, IFNG, IL1A,* and *IL7* were tested by qRT-PCR to verify the expression of genes detected by microarray (Fig. 6). The transcriptional changes in these genes as assessed by qRT-PCR showed similar patterns when compared with the original microarray data, although the magnitude of the response of those genes varied from one method to another.

## Discussion

### General

Although pulmonary intravascular macrophages have been also suggested to support PRRSV replication, the fully developed porcine alveolar macrophages are commonly considered as the cell target for PRRSV infection [17]. The present study investigated the genomic alterations of alveolar macrophages by PRRSV infection and by supplementation of plant extracts to weaned pig feed by microarray analysis that was validated with qRT-PCR. In general, PRRSV infection remarkably affected gene expression in porcine alveolar macrophages at d 14 PI. Dietary supplementation of capsicum oleoresin, garlic botanical, or turmeric oleoresin exhibited different effects on the gene expression in alveolar macrophages isolated from PRRSV-infected pigs. However, the impacts of plant extracts on alveolar macrophages were limited, in comparison to the effects of PRRSV infection. The changes in the expression of immune related genes may help us understand the mechanisms through which plant extracts strengthened immune responses of PRRSV-infected pigs [7].

### Host gene response to PRRSV infection

The course of PRRSV infection can be divided into three stages: 1) acute infection characterized by systemic infection centered in the lung and lymphoid tissues, viremia, and seroconversion; 2) chronic persistent infection characterized by declining antibody titers and declining levels of viral replication in lymphoid tissues; and 3) clearance of infectious virus by immune mechanisms [3, 4]. PRRSV has a complex interaction with the immune system because the primary targets of PRRSV are immune cells. It is important to keep in mind that the present study investigated the gene expression profiles of alveolar macrophages at d 14 PI, which may represent the transition from first stage to second stage of PRRSV infection, because viremia declined but PRRSV antibody titer was increased in the PRRSV-infected pigs relative to earlier stages PI [7].

The innate immune responses of the host against PRRSV involve alveolar macrophages and other immune cells, such as dendritic cells and natural killer cells in the lung. The innate immune responses are critically important for regulating the initiation of viral infection and adaptive immunity. Results of the current study observed that PRRSV infection reduced the expression of several genes related to the innate immune responses in alveolar macrophages, such as, *CD46*, *DDX58*, *FCN2*, *MyD88*, *TLR4*, and *TLR8*. In this gene list, CD46, a type I transmembrane glycoprotein initially identified as a complement inhibitor, has a vital role in the regulation of T cells and complement system [18, 19]. *DDX58* encodes the caspase recruitment domain family RNA helicases RIG-I, which serves as a critical link between toll-like receptor (i.e. TLR8) and type II IFN signaling pathways in antiviral immune responses [20, 21]. The down-regulation of *CD46* and *DDX58* modified the gene expression profiles involved in complement and coagulation cascades. We observed that the expression of *A2M* and *C5* was reduced, whereas the expression of *THBD*, *SERPING1*, *PLAU*, and *F5* was increased in PRRSV-infected alveolar macrophages. The activation of the complement system during viral infection is important for supporting the effectiveness of immune responses and virus neutralization [22]. A large number of complement factors are involved in the complement system/pathway with some of them playing positive regulatory roles but others not. For example, C5 is a strong chemoattractant for neutrophils’ recruitment and activation, whereas A2M plays important roles in cellular activation and signaling transduction in the lung [23–25]. In contrast, *THBD* encodes an endothelial glycoprotein that is highly involved in the inactivation of C3b [26]. *SERPING1* encodes a C1-inihibitor, whose major function is to inhibit complement system in order to prevent spontaneous activation [27]. The downregulation of *A2M* and *C5* and the upregulation of *THBD* and *SERPING1* are highly correlated with the dysfunction of innate immune responses caused by PRRSV infection. Consistently, we also observed that PRRSV infection may reduce alveolar macrophage apoptosis by reducing the expression of *CASP1*, *CASP3*, and *CASP8*. Many viruses inhibit cell apoptosis to prevent premature cell death and thus increase viral replication [28]. Although it has been reported that PRRSV infection induced the apoptosis of infected macrophages at the early stage of the infection, thus delaying the development of adaptive immune responses in PRRSV-infected pigs [29–31]. However, the results of current study indicate that at d 14 PI, alveolar macrophages may move into the anti-apoptotic stage, functioning as normal antigen presenting cells. These results were confirmed by the increased expression of genes involved in antigen presentation (i.e. *MHCI, IFI30, IFIT1, PSMB10*) and the up-regulated expression of cell surface receptors and co-stimulators (i.e. *KLRK1, CCR2, CCR5, CXCR4, IL7R, CD3D, CD59, CD69, CD80,* and *CD247*; Fig. 1). The results are also consistent with the trends of systemic immune responses, as indicated that PRRSV infection increased the peripheral blood leukocyte populations at d 14 PI but not at d 7 PI [7].

PRRSV infection also impacted the mRNA expression of several cytokines in alveolar macrophages, including the up-regulation of *TNFA, IL16, IL18, IL1A, IL10*, *IFNG* and *TGFB1*, and the down-regulation of *IL7, IL15,* and *TGFA1*. The increased expression of *TNFA* and *IL10* are consistent with the serum cytokine results reported by Liu et al. [7] and are likely due to the important regulatory roles of these cytokines in the early immune response to a viral infection [32, 33]. The up-regulation of *IL16* and *IL1A* expression was indicated to be highly involved in lung lesions induced by PRRSV infection [34, 35]. The IFN family, including IFN-α, IFN-β, IFN-γ, is often considered as the first line of defense against viral infections [36]. At the transcriptional level, PRRSV increased *IFNG* expression in alveolar macrophages at d 14 PI, but not *IFNA* and *IFNB*. This result is in close agreement with Barranco et al. [37], who observed that the concentrations of PRRSV antigen and serum IFN-γ displayed a similar bimodal expression trend with a first peak at d 3 to 7 PI and a second peak at d 14 PI, but this was not the case for IFN-α. Several pathways, such as NF-κB pathway, MAPK pathway, and Janus Kinase-Signal Transducer and Activator of Transcription pathway, may be involved in PRRSV infection, depending on the strain of PRRSV and the stage of infection [38, 39]. In the present study, PRRSV infection modified signaling transduction by reducing several genes involved in MAPK signaling pathway but remarkably enhancing the Kegg Pathway of T cell receptor signaling pathway (i.e. *CD247, CD3D, CD3E, CD3G, CD8A*) in alveolar macrophages at d 14 PI. T cell receptor signaling has a central role in response to antigen recognition and in the adaptive immune response [40]. Taken altogether, microarray results indicated that the PRRSV-infected pigs have moved to the stage of the development of adaptive immune response, although it has been suggested that this stage was much delayed by PRRSV compared with other viruses.

Another interesting finding in the current study was PRRSV infection up-regulated the expression of genes (*ADIPOQ, Cpt1b, FABP4, FABP5, LP1, Pltp,* and *scd*; Additional file 1: Supplementary Table 2) involved in PPAR signaling pathway. Peroxisome proliferator-activated receptor γ (PPAR-γ) is an important molecular switch that regulates glucose and lipid metabolism, inflammation, and immunity [41, 42]. This nuclear hormone receptor could antagonize core inflammatory pathways such as NF-κB, AP1, and STAT, therefore, stimulating anti-inflammatory responses during respiratory viral infections [43]. There is limited *in vivo* research reported on PPAR pathway and PRRSV infection. However, one *in vitro* cell culture assay revealed that increased expression of PPAR-γ may be highly involved in the macrophage polarization during PRRSV replication and infection [44]. In addition, two important fatty acid binding proteins (FABPs 4 and 5) are associated with the PPAR pathway and its specific regulatory functions. FABPs are intracellular lipid chaperones, which regulate lipid trafficking and responses in cells [45]. Although FABPs 4 and 5 are mainly expressed in adipocytes, they also exist in macrophages. It has been reported that the expression of FABP4 was increased in macrophages when they were treated with endotoxins, PPAR-γ agonists, and toll-like receptor agonists [46, 47]. Thus, FABPs are also involved in the regulation of inflammatory responses in macrophages [45, 48, 49]. Taken altogether, results in the current study suggested that PPAR pathway was up-regulated by PRRSV infection at d 14 PI, and this up-regulation may enhance anti-inflammatory responses and reduce the risk of secondary infection of PRRSV-infected pigs.

### Plant extracts on PRRSV infection

The anti-inflammatory effects of the same capsicum oleoresin, garlic botanical, and turmeric oleoresin, used in the present experiment, have been confirmed *in vitro* with porcine alveolar macrophages [9]. Our *in vivo* PRRSV challenge trial also suggested that individual supplementation of these plant extracts enhanced immune responses and growth of weaned pigs to different extents, although low dose (10 mg/kg) of plant extracts was used [7]. Consistently, porcine alveolar macrophages in the present study also exhibited different gene expression patterns when they were collected from pigs fed with different plant extracts. Overall, garlic botanical has stronger *in vivo* influences on porcine alveolar macrophages than the two oleoresins.

Supplementation of capsicum oleoresin increased the expression of some immune genes (*A2M, B2M, CREB1, HSP70, HSP90AA1,* and *SLA-1*), but reduced the expression of other immune related genes (*BCL2L1, BID, CASP3, CTNNB1, CCL21, CCR2, CD3D, FASLG,* and *IL1A*) of PRRSV-infected alveolar macrophages. The majority of up-regulated genes were associated with antigen processing and presentation. For example, *B2M* encodes β2-microglobulin, which can form a heterotrimeric complex with the SLA-1 heavy chain, regulating SLA-1 antigen presentation [50]. *HSP70* and *HSP90AA1* encode heat shock proteins that are related to stress response and are linked with innate immune stimulation [51, 52]. Heat shock proteins have been reported to stimulate nonspecific cytokine and chemokine secretion and to activate antigen-presenting cells via a number of cell surface receptors [53, 54]. Moreover, feeding capsicum oleoresin further reduced the expression of genes (*BCL2L1*, *BID*, and *CASP3*) related to cell apoptosis in alveolar macrophages of PRRSV-infected pigs, which may assist in controlling PRRSV replication in macrophages [55]. In combination with the results of lower viral load, the present microarray data suggest that feeding capsicum oleoresin may alleviate the negative effects of PRRSV on innate immunity of weaned pigs.

Compared with capsicum oleoresin, garlic botanical and turmeric oleoresin have greater impacts on the gene expression of alveolar macrophages in PRRSV-infected pigs at d 14 PI. In particular, supplementation of garlic botanical or turmeric oleoresin to PRRSV-infected pigs reduced the mRNA expression of genes associated with antigen processing and presentation pathway in alveolar macrophages (Additional file 1: Supplementary Tables 4 and 5). For example, the mRNA expression of SLA class I and II antigens, *ifi30, SLA-3, SLA-DMB, SLA-DQB, SLA-DRB1, LOC100153090 (Cathepsin S)* were down-regulated in alveolar macrophages collected from pigs supplemented with garlic botanical or turmeric oleoresin. However, pigs supplemented with garlic botanical or turmeric oleoresin were observed to have greater number of B cells and CD8+ T cells on d 14 PI, compared with control pigs [7]. The classic antigen presentation pathway suggests that any form of virus can be presented on MHCs I and II, therefore, stimulating antiviral responses by the activation of both CD8+ and CD4+ T cells [56]. The *in vivo* antigen presentation involves several different cell types, including macrophages, dendritic cells, and other cell types [57]. Results of flow cytometry [7] and microarray indicate that pigs supplemented with garlic botanical or turmeric oleoresin had enhanced cellular responses possibly through other antigen presenting pathways.

With the exception of regulating antigen processing and presentation in alveolar macrophages, administration of garlic botanical also up-regulated the expression of heat shock proteins (*HSP70* and *HSP90AA1*), which are responsible for regulating many transcription factors and kinases implicated in the response to DNA damage stimulus and cell division [58]. Garlic botanical supplementation reduced the expression of *CCL21*, a potent chemoattractant for T cells and dendritic cells and a key instigator of adaptive immune responses [59–61]. In addition, garlic botanical also down-regulated lysosome expression and polysaccharide binding pathways in alveolar macrophages, which are consistent with the reduced antigen processing and presentation in these cells. Both lysosomes and polysaccharide binding receptors are important for maintaining cellular homeostasis and inducing macrophage [62–64].

We also observed that turmeric oleoresin supplementation modulated the expression of other immune related genes (UP: *C5*, *CASP3*, *CASP8*, *IFIT2*, *JAK2*, and TLR8; DOWN: *CCL21*, *IFI30*, *IL1A*, and *PSMB10*). Among these genes, *IFIT2* encodes an important IFN-induced protein, one of the key molecules in innate antiviral immune responses, which may contribute to preventing viral infection and pathogenesis in pigs [65, 66]. Moreover, microarray results indicate that turmeric oleoresin supplementation may affect the calcium signaling pathway in alveolar macrophages. Calcium is one of the critical signaling molecules regulating various functions in cells [67]. Xu et al. [68] reported that curcumin could induce the apoptosis of lung cancer cells through calcium overload. Similar to garlic botanical, turmeric oleoresin supplementation also reduced molecular function of polysaccharide binding in alveolar macrophages. Taken together, garlic botanical and turmeric oleoresin may have similar mechanisms for regulating immune responses of PRRSV-infected alveolar macrophages, in comparison to capsicum oleoresin.

## Conclusions

As one of the key players in immunoregulation, the infection of alveolar macrophages challenges both innate and adaptive immunity of PRRSV-infected pigs. Although much research has deciphered the transcriptome profile of lung immune cells (i.e. macrophages or dendritic cells) *in vitro* and *in vivo*, this is the first experiment reporting the impacts of PRRSV on the gene expression profiles of alveolar macrophages that were isolated from pigs 2 weeks post inoculation. As discussed above, these PRRSV-infected pigs may have already passed the immune suppressive stage and moved to the second phase of PRRSV infection. During this stage, the apoptosis of macrophages was reduced, and the development of adaptive immunity was increased, as indicated by enhanced antigen processing and presentation, T cell signaling transduction, and inflammatory cytokine expression.

At the transcriptional level, supplementation of three plant extracts had varied impacts on alveolar macrophages isolated from PRRSV infection pigs, with garlic botanical and turmeric oleoresin sharing more similarity. Feeding garlic botanical and turmeric oleoresin down-regulated the mRNA abundance of genes related to antigen processing and polysaccharide binding, while feeding capsicum oleoresin up-regulated the expression of genes associated with antigen processing and presentation. Moreover, feeding capsicum oleoresin, garlic botanical, and turmeric oleoresin also differently altered the gene expression related to cell apoptosis, intracellular signaling transduction, and other biological processes. Although the impacts of plant extracts on alveolar macrophages are limited, the majority of findings are consistent with the data of performance and plasma measurements in pigs. Overall, garlic botanical has stronger *in vivo* infuences on porcine alveolar macrophages than the two oleoresins, whereas turmeric oleoresin had stromger impacts on growth efficiency and overall immune responses of weaned pigs. Therefore, combing different plant extracts may exhibit add-up benefits on performance and disease resistance of weaned pigs.

## Supplementary information

**Additional file 1: Supplementary Table 1.** Gene-specific primer sequences and PCR conditions. **Supplementary Table 2**. Modulation of KEGG pathway and biological processes in alveolar macrophages by PRRSV infection when pigs fed the control diet. **Supplementary Table 3**. Supplementation of capsicum oleoresin to PRRSV-infected pigs modulates KEGG pathway in alveolar macrophage of weaned pigs. **Supplementary Table 4**. Supplementation of garlic botanical to PRRSV-infected pigs modulates KEGG pathway and biological process in alveolar macrophage of weaned pigs. **Supplementary Table 5**. Supplementation of turmeric oleoresin to PRRSV-infected pigs modulates KEGG pathway in alveolar macrophage of weaned pigs.

## Data Availability

All data generated or analyzed during this study are available from the corresponding author upon reasonable request.

## Abbreviations

ACTB: Beta actin
BW: Body weight
CASP3: Caspase 3
CCL5: Chemokine ligand 5
EASE: Expression analysis systemic explorer
GAPDH: Glyceraldehyde 3-phosphate dehydrogenase
IFNG: Interferon gamma
IL1A: Interleukin 1 alpha
IL7: Interleukin 7
KEGG: Kyoto Encyclopedia of Genes and Genomes
MAPK: Mitogen-activated protein kinase
MHC: Major histocompatibility complex
PBS: Phosphate buffer saline
PI: Post-inoculation
PPAR: Peroxisome proliferator-activated receptor
PRRSV: Porcine reproductive and respiratory syndrome virus
qRT-PCR: Quantitative real time-polymerase chain reaction
TGF-beta: Transforming growth factor-beta
