# Supplementary material for: Dietary plant extracts modulate gene expression profiles in alveolar macrophages of pigs experimentally infected with porcine reproductive and respiratory syndrome virus
Source: J Anim Sci Biotechnol. 2020 Jul 14;11:74. doi: 10.1186/s40104-020-00475-w (PMC7359597; doi:10.1186/s40104-020-00475-w)
Supplement: Supplementary file 1 — Additional file 1: Supplementary Table 1. Gene-specific primer sequences and PCR conditions. Supplementary Table 2. Modulation of KEGG pathway and biological processes in alveolar macrophages by PRRSV infection when pigs fed the control diet. Supplementary Table 3. Supplementation of capsicum oleoresin to PRRSV-infected pigs modulates KEGG pathway in alveolar macrophage of weaned pigs. Supplementary Table 4. Supplementation of garlic botanical to PRRSV-infected pigs modulates KEGG pathway and biological process in alveolar macrophage of weaned pigs. Supplementary Table 5. Supplementation of turmeric oleoresin to PRRSV-infected pigs modulates KEGG pathway in alveolar macrophage of weaned pigs. [file 40104_2020_475_MOESM1_ESM.docx]

**Online-Only Data Supplements**

**Supplementary Table 1**. Gene-specific primer sequences and PCR conditions^1^

| Gene^2^ | Acc. No^3^ | Forward primer (5’→3’) | Reverse primer (5’→3’) |
| --- | --- | --- | --- |
| *CASP3* | NM_214131.1 | ATTCAGGCCTGCCGAGGCAC | CCCACTGTCCGTCTCAATCCCA |
| *CCL5* | NM_001129946.1 | CATGGCAGCAGTCGTCTTTA | AAGGCTTCCTCCATCCTAGC |
| *IFNG* | NM_213948.1 | TCTAACCTAAGAAAGCGGAAGAGAA | TTGCAGGCAGGATGACAATTA |
| *IL1A* | NM_214029.1 | CAGCCAACGGGAAGATTCTG | ATGGCTTCCAGGTCGTCAT |
| *IL7* | NM_214135.1 | CAACTGCACCAGCAAGGTTAAAG | AAGTCCCCCTGTCTTTTCTGTTC |
| *ACTB* | DQ178122 | TCTGGCACCACACCTTCT | TGATCTGGGTCATCTTCTCAC |
| *GAPDH* | [NM_214353](http://www.sciencedirect.com/science?_ob=RedirectURL&_method=externObjLink&_locator=genbank&_issn=03784274&_origin=article&_zone=art_page&_plusSign=%2B&_targetURL=http%253A%252F%252Fwww.ncbi.nlm.nih.gov%252Fentrez%252Fquery.fcgi%253Fcmd%253Dsearch%2526db%253Dnucleotide%2526doptcmdl%253Dgenbank%2526term%253DNM_214353%5baccn%5d) | ATAAGTGTGACTGCACCCGAAC | GGTGGGCTATCAATCAGATGTG |

^1^Thermal cycling conditions were 50°C for 2 min and 95°C for 10 min, followed by 40 cycles with 15 sec at 95°C and 1 min at 60°C.

^2^*CASP3*: caspase 3; *CCL5*: chemokine (C-C motif) ligand 5; *IFNG*: interferon-gamma.

^3^Accession number in GenBank database.

**Supplementary Table 2**. Modulation of KEGG pathway and biological processes in alveolar macrophages by PRRSV infection when pigs fed the control diet^1^

| Category | Entrez ID | Gene symbol | Gene name | Fold change^2^ |
| --- | --- | --- | --- | --- |
| T cell receptor signaling pathway (KEGG pathway) | | | |  |
|  | 397302 | CD247 | CD247 molecule | 23.92 |
|  | 396661 | CD3D | CD3d molecule, delta (CD3-TCR complex) | 40.03 |
|  | 397455 | CD3E | CD3e molecule, epsilon (CD3-TCR complex) | 54.99 |
|  | 494013 | CD3G | CD3g molecule, gamma (CD3-TCR complex) | 53.04 |
|  | 396627 | CD8A | CD8a molecule | 101.48 |
|  | 396636 | CD8B | CD8b molecule | 91.96 |
|  | 791125 | FYN | FYN oncogene related to SRC, FGR, YES | 2.39 |
|  | 396991 | IFNG | Interferon-gamma | 56.81 |
|  | 397106 | IL10 | Interleukin 10 | 11.38 |
|  | 100158082 | LOC100158082 | Similar to protein phosphatase 3, catalytic subunit, gamma | 2.55 |
|  | 397086 | TNF | Tumor necrosis factor (TNF superfamily, member 2) | 2.16 |
| Cytokine-cytokine receptor interaction (KEGG Pathway) | | | |  |
|  | 494459 | CCL3L1 | Chemokine (C-C motif) ligand 3-like 1 | 61.99 |
|  | 396668 | CCL4 | Chemokine (C-C motif) ligand 4 | 83.98 |
|  | 396613 | CCL5 | Chemokine (C-C motif) ligand 5 | 78.41 |
|  | 414371 | CCR5 | Chemokine (C-C motif) receptor 5 | 60.17 |
|  | 100135681 | CXCL9 | Chemokine (C-X-C motif) ligand 9 | 110.81 |
|  | 414376 | CXCR6 | Chemokine (C-X-C motif) receptor 6 | 101.33 |
|  | 396991 | IFNG | Interferon-gamma | 56.81 |
|  | 397106 | IL10 | Interleukin 10 | 11.38 |
|  | 397057 | IL18 | Interleukin 18 (interferon-gamma-inducing factor) | 6.91 |
|  | 100155581 | LOC100155581 | Lymphotoxin beta | 14.08 |
|  | 397086 | TNF | Tumor necrosis factor (TNF superfamily, member 2) | 2.16 |
| Complement and coagulation cascades (KEGG pathway) | | | |  |
|  | 100157642 | THBD | Thrombomodulin | 7.14 |
|  | 100144304 | SERPING1 | Serpin peptidase inhibitor clade G (C1 inhibitor) member 1 | 4.86 |
|  | 396985 | PLAU | Plasminogen activator | 3.50 |
|  | 396677 | LOC396677 | Tissue factor | 3.37 |
|  | 100153504 | LOC100153504 | Similar to coagulation factor XIII A1 subunit | 83.4 |
|  | 397217 | F5 | Coagulation factor V | 2.13 |
| PPAR signaling pathway (KEGG pathway) | | | |  |
|  | 397660 | ADIPOQ | Adiponectin | 5.85 |
|  | 399528 | CPT1B | Carnitine palmitoyltransferase 1B (muscle) | 2.91 |
|  | 399533 | FABP4 | Fatty acid binding protein 4 | 8.78 |
|  | 574074 | FABP5 | Fatty acid binding protein 5 | 2.93 |
|  | 397537 | LPL | Lipoprotein lipase | 16.11 |
|  | 397527 | PLTP | Phospholipid transfer protein | 4.28 |
|  | 396670 | SCD | Stearoyl-CoA desaturase | 2.38 |
| TGF-beta signaling pathway (KEGG pathway) | | | |  |
|  | 100152307 | ACVR1 | Similar to activin A type I receptor | -2.92 |
|  | 100152069 | LOC100152069 | Similar to Mothers against decapentaplegic homolog 6 (SMAD 6) (Mothers against DPP homolog 6) (Smad6) (hSMAD6) | -3.35 |
|  | 100153524 | LOC100153524 | Similar to bone morphogenetic protein receptor, type IA | -3.71 |
|  | 448810 | MYC | v-myc myelocytomatosis viral oncogene homolog (avian) | -2.73 |
|  | 397016 | SMAD1 | SMAD family member 1 | -2.74 |
|  | 396665 | TGFBR1 | Transforming growth factor beta receptor 1 | -4.00 |
|  | 492313 | THBS1 | Thrombospondin 1 | -89.57 |
| MAPK signaling pathway (KEGG pathway) | | | |  |
|  | 397244 | CASP3 | Caspase 3, apoptosis-related cysteine peptidase | -3.98 |
|  | 397070 | EGFR | Epidermal growth factor receptor | -6.39 |
|  | 100144486 | FOS | v-fos FBJ murine osteosarcoma viral oncogene homolog | -3.02 |
|  | 100152997 | GADD45G | Similar to growth arrest and DNA-damage-inducible gamma | -2.82 |
|  | 396906 | HSP70 | Heat shock protein 70 | -2.92 |
|  | 396648 | HSP70.2 | Heat shock protein 70.2 | -5.23 |
|  | 397094 | IL1A | Interleukin 1, alpha | -7.81 |
|  | 100152046 | LOC100152046 | Similar to ribosomal protein S6 kinase, 90kDa, polypeptide 5 | -3.38 |
|  | 100155841 | LOC100155841 | Similar to mitogen-activated protein kinase 13 | -2.01 |
|  | 100156630 | LOC100156630 | Similar to mitogen-activated protein kinase 14 | -2.35 |
|  | 448810 | MYC | v-myc myelocytomatosis viral oncogene homolog (avian) | -2.73 |
|  | 396665 | TGFBR1 | Transforming growth factor | -4.00 |
| Response to stimulus (Biological process) | | | |  |
|  | 396900 | AMCF-II | Alveolar macrophage-derived chemotactic factor-II | 5.10 |
|  | 397033 | B2M | Beta-2-microglobulin | 2.31 |
|  | 397422 | CCL2 | Chemokine (C-C motif) ligand 2 | 221.63 |
|  | 448797 | CCL21 | Chemokine (C-C motif) ligand 21 | 2.78 |
|  | 494459 | CCL3L1 | Chemokine (C-C motif) ligand 3-like 1 | 61.99 |
|  | 396668 | CCL4 | Chemokine (C-C motif) ligand 4 | 83.98 |
|  | 396613 | CCL5 | Chemokine (C-C motif) ligand 5 | 78.41 |
|  | 396785 | CD1.1 | CD1 antigen | 11.03 |
|  | 100135681 | CXCL9 | Chemokine (C-X-C motif) ligand 9 | 110.81 |
|  | 397217 | F5 | Coagulation factor V | 2.13 |
|  | 396991 | IFNG | Interferon-gamma | 56.81 |
|  | 397106 | IL10 | Interleukin 10 | 11.38 |
|  | 397057 | IL18 | Interleukin 18 (interferon-gamma-inducing factor) | 6.91 |
|  | 100155581 | LOC100155581 | Lymphotoxin beta | 14.08 |
|  | 396677 | LOC396677 | Tissue factor | 3.37 |
|  | 396877 | LOC396877 | Complement factor D | 6.25 |
|  | 733603 | LOC733603 | Serum amyloid A2 | 11.12 |
|  | 780420 | LOC780420 | SH21A | 159.79 |
|  | 397538 | ME1 | Malic enzyme 1, NADP(+)-dependent, cytosolic | 2.60 |
|  | 396869 | NKL | NK-lysin | 225.66 |
|  | 100135037 | SLA-6 | MHC class I antigen 6 | 2.31 |
|  | 397078 | TGFB1 | Transforming growth factor, beta 1 | 2.72 |
|  | 100157642 | THBD | Thrombomodulin | 7.14 |
|  | 396621 | TLR6 | Toll-like receptor 6 | 3.96 |
|  | 397007 | TLR9 | Toll-like receptor 9 | 2.28 |
|  | 397086 | TNF | Tumor necrosis factor (TNF superfamily, member 2) | 2.16 |
| Cell cycle (Biological process) | | |  |  |
|  | 396955 | AURKB | Aurora kinase B | 2.87 |
|  | 397266 | BIRC5 | Baculoviral IAP repeat-containing 5 | 3.08 |
|  | 100135668 | CCNB2 | Cyclin B2 | 2.32 |
|  | 397162 | CCND2 | Cyclin D2 | 30.36 |
|  | 397379 | CDC20 | Cell division cycle 20 homolog (S. cerevisiae) | 2.91 |
|  | 733584 | FOXN3 | Forkhead box N3 | 2.09 |
|  | 733670 | RGS2 | Regulator of G-protein signaling 2, 24kDa | 21.30 |
|  | 733608 | S100A6 | S100 calcium-binding protein A6 | 6.19 |
| Cell adhesion (Biological process) | | |  |  |
|  | 396662 | CD2 | CD2 molecule | 68.03 |
|  | 397217 | F5 | Coagulation factor V | 2.13 |
|  | 397063 | ITGB3 | Integrin, beta 3 (platelet glycoprotein IIIa, antigen CD61) | 12.55 |
|  | 396941 | PECAM1 | Platelet/endothelial cell adhesion molecule | 13.78 |
|  | 397018 | SCARB1 | Scavenger receptor class B, member 1 | 16.77 |
|  | 100127147 | SELL | Selectin L | 14.7 |
|  | 397087 | SPP1 | Secreted phosphoprotein 1 | 7.72 |
|  | 396925 | VCAM1 | Vascular cell adhesion molecule | 5.91 |
| Signal transduction (Biological process) | | | |  |
|  | 397628 | ANGPTL4 | Angiopoietin-like 4 | -10.13 |
|  | 414374 | CCR1 | Chemokine (C-C motif) receptor 1 | -2.07 |
|  | 397243 | CYSLT2 | CYSLT2 protein | -7.02 |
|  | 397060 | DUOX2 | Dual oxidase 2 | -7.29 |
|  | 100037272 | EPAS1 | Endothelial PAS domain protein 1 | -5.36 |
|  | 396881 | FCN2 | Ficolin | -4.22 |
|  | 448808 | FGL2 | Fibrinogen-like 2 | -3.07 |
|  | 422408 | GUCY1B3gucy1b3 | Guanylate cyclase 1, soluble, beta 3 | -7.66 |
|  | 396696 | HIF1A | Hypoxia inducible factor 1, alpha subunit (basic helix-loop-helix transcription factor) | -3.76 |
|  | 595121 | LOC595121 | ADP-ribosylation factor-like protein 4A | -27.9 |
|  | 396646 | MYD88 | Myeloid differentiation primary response gene (88) | -2.16 |
|  | 574068 | NCOA1 | Nuclear receptor coactivator 1 | -2.06 |
|  | 100135658 | NCOA2 | Nuclear receptor coactivator 2 | -2.15 |
|  | 595117 | RAB11A | Ras-related protein Rab-11A | -2.83 |
|  | 100144496 | RAB32 | RAB32, member RAS oncogene family | -2.01 |
|  | 100144500 | RHOF | Ras homolog gene family, member F | -2.94 |
|  | 100141401 | S1PR5 | Sphingosine-1-phosphate receptor 5 | -4.11 |
|  | 397151 | SAG | S-antigen visual arrestin | -6.26 |
|  | 397484 | TGFA | Transforming growth factor, alpha | -8.44 |
|  | 399541 | TLR4 | Toll-like receptor 4 | -8.26 |
|  | 397384 | TLR8 | Toll-like receptor 8 | -3.19 |
| Positive regulation of biological process (Biological process) | | | |  |
|  | 397568 | CAT | Catalase | -2.23 |
|  | 396922 | CD46 | CD46 molecule, complement regulatory protein | -2.50 |
|  | 396723 | DDX58 | DEAD (Asp-Glu-Ala-Asp) box polypeptide 58 | -3.29 |
|  | 396881 | FCN2 | Ficolin | -4.22 |
|  | 397028 | HSP90AA1 | 90-kDa heat shock protein | -2.25 |
|  | 448812 | IGFBP3 | Insulin-like growth factor binding protein 3 | -8.54 |
|  | 397094 | IL1A | Interleukin 1, alpha | -7.81 |
|  | 448810 | MYC | v-myc myelocytomatosis viral oncogene homolog (avian) | -2.73 |
|  | 397671 | PPARG | Peroxisome proliferator-activated receptor gamma | -3.77 |
|  | 396870 | PPBP | Platelet basic protein | -8.61 |
|  | 397484 | TGFA | Transforming growth factor, alpha | -8.44 |
|  | 397384 | TLR8 | Toll-like receptor 8 | -3.19 |
| Innate immune response (Biological process) | | | |  |
|  | 396922 | CD46 | CD46 molecule, complement regulatory protein | -2.50 |
|  | 396723 | DDX58 | DEAD (Asp-Glu-Ala-Asp) box polypeptide 58 | -3.29 |
|  | 396881 | FCN2 | Ficolin | -4.22 |
|  | 396646 | MYD88 | Myeloid differentiation primary response gene (88) | -2.16 |
|  | 399541 | TLR4 | Toll-like receptor 4 | -8.26 |
|  | 397384 | TLR8 | Toll-like receptor 8 | -3.19 |

^1^All data were analyzed by DAVID Bioinformatics Resources 6.7 (National Institute of Allergy and Infectious Diseases (NIAID, NIH)). PRRSV = porcine reproductive and respiratory syndrome virus.

^2^Negative value indicates reduction in gene expression.

**Supplementary Table 3**. Supplementation of capsicum oleoresin to PRRSV-infected pigs modulates KEGG pathway in alveolar macrophage of weaned pigs^1^

| Category | Entrez ID | Gene symbol | Gene name | Fold change^2^ |
| --- | --- | --- | --- | --- |
| Antigen processsing and presentation (KEGG pathway) | | | |  |
|  | 397033 | B2M | Beta-2-microglobulin | 1.62 |
|  | 397449 | CREB1 | cAMP responsive element binding protein 1 | 1.53 |
|  | 397028 | HSP90AA1 | 90-kDa heat shock protein | 1.52 |
|  | 100153090 | LOC100153090 | Similar to Cathepsin S | 1.92 |
|  | 100037293 | SLA-1 | MHC class I antigen 1 | 1.82 |
| Steroid hormone biosynthesis (KEGG pathway) | | | |  |
|  | 403322 | CYP3A39 | Cytochrome P450 3A39 | 2.18 |
|  | 100147712 | HSD17B1 | Hydroxysteroid (17-beta) dehydrogenase 1 | 1.63 |
|  | 397052 | SULT1E1 | Sulfotransferase family 1E, estrogen-preferring, member 1 | 1.54 |
| Pathways in cancer (KEGG pathway) | | |  |  |
|  | 397536 | BCL2L1 | BCL2-like 1 | -1.94 |
|  | 594852 | BID | BH3 interacting domain death agonist | -1.85 |
|  | 397244 | CASP3 | Caspase 3, apoptosis-related cysteine peptidase | -1.51 |
|  | 397657 | CTNNB1 | Catenin (cadherin-associated protein) beta 1, 88 kDa | -1.89 |
|  | 396726 | FASLG | Fas ligand (TNF superfamily member 6) | -1.76 |
|  | 100153927 | LOC100153927 | Similar to Mitogen-activated protein kinase 1 (Extracellular signal-regulated kinase 2) (ERK-2) (Mitogen-activated protein kinase 2) (MAP kinase 2) (MAPK 2) (p42-MAPK) (ERT1) | -1.59 |
|  | 100155896 | LOC100155896 | Similar to coiled-coil domain containing 6 | -1.52 |

^1^All data were analyzed by DAVID Bioinformatics Resources 6.7 (National Institute of Allergy and Infectious Diseases (NIAID, NIH)). PRRSV = porcine reproductive and respiratory syndrome virus.

^2^Negative value indicates reduction in gene expression.

**Supplementary Table 4**. Supplementation of garlic botanical to PRRSV-infected pigs modulates KEGG pathway and biological process in alveolar macrophage of weaned pigs^1^

| Category | Entrez ID | Gene symbol | Gene name | Fold change^2^ |
| --- | --- | --- | --- | --- |
| Glycine, serine and threonine metabolism (KEGG pathway) | | | |  |
|  | 397129 | DLD | Dihydrolipoamide dehydrogenase | 1.60 |
|  | 100152340 | LOC100152340 | Similar to Serine hydroxymethyltransferase 2 (mitochondrial) | 1.50 |
|  | 100154160 | LOC100154160 | Similar to phosphoserine aminotransferase | 15.22 |
|  | 100144529 | PHGDH | Phosphoglycerate dehydrogenase | 5.98 |
| Antigen processing and presentation; Intestinal immune network for IgA production; Cell adhesion molecules (KEGG pathway*)* | | | | |
|  | 100127162 | TNFSF13 (APRIL) | TNF sperfamily member 13 (A proliferation-inducing ligand) | -2.01 |
|  | 396702 | CD58 | CD58 molecule | -1.86 |
|  | 396660 | CD74 | CD74 antigen | -1.89 |
|  | 100174943 | IFI30 | Interferon, gamma-inducible protein 30 | -1.69 |
|  | 396943 | ITGB2 | Integrin, beta 2 (complement component 3 receptor 3 and 4 subunit) | -1.61 |
|  | 100152754 | LOC100152754 | similar to syndecan 2 | -1.51 |
|  | 100153090 | LOC100153090 | Similar to Cathepsin S | -1.57 |
|  | 100154477 | LOC100154477 | Similar to legumain | -1.84 |
|  | 100037288 | SLA-3 | MHC class I antigen 3 | -1.53 |
|  |  |  |  |  |
|  | 100135036 | SLA-8 | MHC class I antigen 8 | -1.51 |
|  | 100135050 | SLA-DMB | MHC class II, DM beta | -1.57 |
|  | 100135038 | SLA-DOA | Major histocompatibility complex, class II, DO alpha | -1.60 |
|  | 100135044 | SLA-DQA | MHC class II, DQ alpha | -1.52 |
|  | 100037921 | SLA-DQB1 | SLA-DQ beta1 domain | -1.53 |
|  | 100153386 | SLA-DRB1 | MHC class II histocompatibility antigen SLA-DRB1 | -1.57 |
|  | 397078 | TGFB1 | Transforming growth factor, beta 1 | -1.61 |
| Cell cycle (Biological process) | | | |  |
|  | 396955 | AURKB | Aurora kinase B | 1.88 |
|  | 397266 | BIRC5 | Baculoviral IAP repeat-containing 5 | 1.64 |
|  | 100144474 | CDC25C | Cell division cycle 25 homolog C (S. pombe) | 1.64 |
|  | 397378 | PPP1CB | Protein phosphatase 1, beta isoform | 1.54 |
|  | 397015 | PTTG1 | Pituitary tumor-transforming 1 | 1.69 |
|  | 733608 | S100A6 | S100 calcium-binding protein A6 catalytic subunit | 1.71 |
| Response to DNA damage stimulus (Biological process) | | | |  |
|  | 445521 | HMGB1 | High-mobility group box 1 | 1.71 |
|  | 396648 | HSP70.2 | Heat shock protein 70.2 | 1.94 |
|  | 397015 | PTTG1 | Pituitary tumor-transforming 1 | 1.69 |
|  | 100217383 | RAD18 | RAD18 homolog (S. cerevisiae) | 1.56 |
| Immune response (Biological process) | | | | |
|  | 100127162 | TNFSF13 (APRIL) | TNF sperfamily member 13 (A proliferation-inducing ligand) | -2.03 |
|  | 445461 | C1QAqa | Complement component 1, A chain | -2.45 |
|  | 448981 | C2 | Complement component 2 | -2.73 |
|  | 397072 | C3 | Complement component 3, class II | -2.60 |
|  | 448797 | CCL21 | Chemokine (C-C motif) ligand 21 | -2.55 |
|  | 396660 | CD74 | CD74 antigen | -1.89 |
|  | 397399 | FCGRT | Fc fragment of IgG transporter, alpha | -1.59 |
|  | 396646 | MYD88 | Myeloid differentiation primary response gene (88) | -1.69 |
|  | 100037288 | SLA-3 | MHC class I antigen 3 | -1.53 |
|  | 100135036 | SLA-8 | MHC class I antigen 8 | -1.51 |
|  | 100135050 | SLA-DMB | MHC class II, DM beta | -1.57 |
|  | 100135038 | SLA-DOA | Major histocompatibility complex, class II, DO alpha | -1.60 |
|  | 100135044 | SLA-DQA | MHC class II, DQ alpha | -1.52 |
|  | 100037921 | SLA-DQB1 | SLA-DQ beta1 domain | -1.53 |
|  | 100153386 | SLA-DRB1 | MHC class II histocompatibility antigen SLA-DRB1 | -1.57 |
|  | 399541 | TLR4 | Toll-like receptor 4 | -1.60 |
|  | 396621 | TLR6 | Toll-like receptor 6 | -2.09 |
|  | 397007 | TLR9 | Toll-like receptor 9 | -2.10 |
| Cell death (Biological process) | | | | |
|  | 397663 | APP | Amyloid beta (A4) precursor protein, member 1A | -1.71 |
|  | 100127160 | CIDEB | Cell-death-inducing DNA-fragmentation-factor-like effector b | -1.80 |
|  | 397398 | DNASE2 | Deoxyribonuclease II, beta 1 | -1.54 |
|  | 641352 | LOC641352 | Caspase-15 | -1.51 |
|  | 397078 | TGFB1 | Transforming growth factor | -1.61 |
|  | 396907 | TMBIM6 | Transmembrane BAX inhibitor motif containing 6 | -1.87 |
|  | 397020 | TNFRSF1A | Tumor necrosis factor receptor superfamily | -1.51 |

^1^All data were analyzed by DAVID Bioinformatics Resources 6.7 (National Institute of Allergy and Infectious Diseases (NIAID, NIH)). PRRSV = porcine reproductive and respiratory syndrome virus.

^2^Negative value indicates reduction in gene expression.

**Supplementary Table 5**. Supplementation of turmeric oleoresin to PRRSV-infected pigs modulates KEGG pathway in alveolar macrophage of weaned pigs^1^

| Category | Entrez ID | Gene symbol | Gene name | Fold change^2^ |
| --- | --- | --- | --- | --- |
| RNA degradation (KEGG pathway) | | | |  |
|  | 492279 | HSPD1 | Heat shock 60 kDa protein 1 (chaperonin) | 1.60 |
|  | 100154807 | LOC100154807 | Similar to 5-3 exoribonuclease 2 | 1.53 |
|  | 100156323 | LOC100156323 | Similar to polynucleotide adenylyltransferase | 1.68 |
|  | 100157236 | LOC100157236 | Similar to Exosome component 8 | 1.77 |
| Antigen processing and presentation; Viral myocarditis (KEGG pathway) | | | | |
|  | 397033 | B2M | Beta-2-microglobulin | -1.50 |
|  | 594852 | BID | BH3 interacting domain death agonist | -1.58 |
|  | 397028 | HSP90AA1 | 90-kDa heat shock protein | -1.65 |
|  | 396750 | ICAM-1 | Intercellular adhesion molecule-1 | -1.50 |
|  | 100174943 | IFI30 | Interferon gamma-inducible protein 30 | -1.62 |
|  | 100153090 | LOC100153090 | Similar to Cathepsin S | -1.62 |
|  | 100135031 | SLA-2 | MHC class I antigen 2 | -1.77 |
|  | 100037288 | SLA-3 | MHC class I antigen 3 | -1.72 |
|  | 100135050 | SLA-DMB | MHC class II DM beta | -1.52 |
|  | 100037921 | SLA-DQB1 | SLA-DQ beta1 domain | -1.51 |
|  | 100153386 | SLA-DRB1 | MHC class II histocompatibility antigen SLA-DRB1 | -1.82 |
| Natural killer cell mediated cytotoxicity (KEGG pathway) | | | |  |
|  | 397267 | HCST | Hematopoietic cell signal transducer | -1.76 |
|  | 396750 | ICAM1 | Intercellular adhesion molecule-1 | -1.50 |
|  | 414387 | ICAM2 | Intercellular adhesion molecule-2 | -1.66 |
|  | 396726 | FASLG | Fas ligand (TNF superfamily, member 6) | -1.77 |
|  | 594852 | BID | BH3 interacting domain death agonist | -1.58 |
|  | 733587 | LOC733587 | v-Ha-ras Harvey rat sarcoma viral oncogene homolog | -1.67 |
|  | 397405 | TYROBP | TYRO protein tyrosine kinase binding protein | -1.52 |

^1^All data were analyzed by DAVID Bioinformatics Resources 6.7 (National Institute of Allergy and Infectious Diseases (NIAID, NIH)). PRRSV = porcine reproductive and respiratory syndrome virus.

^2^Negative value indicates reduction in gene expression.
